# Phytotoxic Tryptoquialanines Produced *In Vivo* by *Penicillium digitatum* Are Exported in Extracellular Vesicles

**DOI:** 10.1128/mBio.03393-20

**Published:** 2021-02-09

**Authors:** Jonas Henrique Costa, Jaqueline Moraes Bazioli, Luidy Darllan Barbosa, Pedro Luis Theodoro dos Santos Júnior, Flavia C. G. Reis, Tabata Klimeck, Camila Manoel Crnkovic, Roberto G. S. Berlinck, Alessandra Sussulini, Marcio L. Rodrigues, Taícia Pacheco Fill

**Affiliations:** a Institute of Chemistry, University of Campinas, CP 6154, Campinas, São Paulo, Brazil; b Faculty of Pharmaceutical Sciences, University of Campinas, Campinas, São Paulo, Brazil; c Instituto Carlos Chagas, Fundação Oswaldo Cruz (Fiocruz), Curitiba, Paraná, Brazil; d Centro de Desenvolvimento Tecnológico em Saúde (CDTS), Fiocruz, Rio de Janeiro, Brazil; e Department of Biochemical and Pharmaceutical Technology, School of Pharmaceutical Sciences, University of São Paulo, São Paulo, São Paulo, Brazil; f Instituto de Química de São Carlos, Universidade de São Paulo, CP 780, São Carlos, São Paulo, Brazil; g Instituto de Microbiologia Paulo de Góes (IMPG), Universidade Federal do Rio de Janeiro, Rio de Janeiro, Rio de Janeiro, Brazil; University of British Columbia

**Keywords:** fungi, extracellular vesicles, herbicidal activity, host-pathogen interaction, *P. digitatum*, tryptoquialanines, *Penicillium digitatum*

## Abstract

Penicillium digitatum is the most aggressive pathogen of citrus fruits. Tryptoquialanines are major indole alkaloids produced by *P. digitatum*. It is unknown if tryptoquialanines are involved in the damage of citrus fruits caused by *P. digitatum.* To investigate the pathogenic roles of tryptoquialanines, we initially asked if tryptoquialanines could affect the germination of Citrus sinensis seeds. Exposure of the citrus seeds to tryptoquialanine A resulted in a complete inhibition of germination and an altered metabolic response. Since this phytotoxic effect requires the extracellular export of tryptoquialanine A, we investigated the mechanisms of extracellular delivery of this alkaloid in *P. digitatum*. We detected extracellular vesicles (EVs) released by *P. digitatum* both in culture and during infection of citrus fruits. Compositional analysis of EVs produced during infection revealed the presence of a complex cargo, which included tryptoquialanines and the mycotoxin fungisporin. The EVs also presented phytotoxicity activity *in vitro* and caused damage to the tissues of citrus seeds. Through molecular networking, it was observed that the metabolites present in the *P. digitatum* EVs are produced in all of its possible hosts. Our results reveal a novel phytopathogenic role of *P. digitatum* EVs and tryptoquialanine A, implying that this alkaloid is exported in EVs during plant infection.

## Abstract (precis)

During the postharvest period, citrus fruits can be affected by phytopathogens such as Penicillium digitatum, which causes green mold disease and is responsible for up to 90% of total citrus losses. Chemical fungicides are widely used to prevent green mold disease, leading to concerns about environmental and health risks.

## INTRODUCTION

Citriculture is a worldwide multi-billion-dollar activity (1). Brazil, China, and the United States are the major producers of citrus (2). In Brazil, 230,000 direct and indirect jobs are related to citriculture (3). The citrus industry in Brazil corresponded to US$6.5 billion in revenues in 2019 (3). Citrus fruits can be affected by different diseases, leading to up to 50% of fruit losses and causing a negative economic impact (4–6).

Diseases caused by fungal pathogens are the most adverse factors causing fresh fruit and vegetable losses during the postharvest period (7, 8). Postharvest losses due to fungal diseases can reach 30 to 55% of production (8–10). The most damaging postharvest disease in citrus is the green mold caused by Penicillium digitatum, which accounts for up to 90% of citrus losses (4–6).

Demethylation inhibitors (DMI), including prochloraz and imazalil, are fungicides used to combat *P. digitatum* (4). However, this practice has raised concerns about its effects on human health and the development of antifungal resistance (10, 11). Therefore, developing safer approaches to control postharvest diseases has become a global trend (7, 8, 10, 11). The development of alternative antifungal tools demands an improved knowledge of how *P. digitatum* causes damage to citrus fruits. Most efforts in this direction have focused on the use of biocontrol agents, antagonist microorganisms, and natural products (8, 11, 12) to neutralize virulence factors (4, 13). Nevertheless, the molecular mechanisms underlying the induction of *P. digitatum*-mediated damage in host cells remain poorly known (4, 5, 14).

Secondary metabolites were reported as essential for fungal pathogenicity and a consequent attenuation of the plant defense responses (4, 5, 14). Siderophores, for instance, have been associated with fungal virulence by iron sequestration (4, 15). Secondary metabolites have not yet been associated with the pathogenic process promoted by *P. digitatum* (4). Tryptoquialanines are major metabolites produced by *P. digitatum* (16). However, their role in *P. digitatum* phytopathogenicity is unclear, even though these compounds displayed insecticidal (17) and antifungal (18) activities.

In addition to secondary metabolites, extracellular vesicles (EVs) have been associated with the pathogenesis of several infectious diseases (19, 20). EVs are spherical structures that are released by bacteria, fungi, and plant cells (19–21). EVs are delimited by a lipid bilayer membrane in association with proteins, lipids, enzymes, pigments, polysaccharides, and RNAs (19–21). In fungi, EVs were first reported in the human-pathogenic yeast Cryptococcus neoformans and subsequently characterized in several fungal pathogens (20, 21). In host-microbe interactions, EVs are key players determining the pathogenic outcome (22, 23) and mediating the transfer of virulence traits (24). In plant infections, the roles of EVs have been superficially explored, and the knowledge of how EVs impact the plant physiology is limited (22, 23). It is known that under stress conditions, release of EVs by plants is increased in response to infection (22, 23). EVs are involved in the defense of plants against pathogens, forming physical barriers or delivering molecules that are toxic to invading microbes (22, 23). On the other hand, pathogen EVs can inhibit plant immune responses through the export of virulence factors (22). So far, no information on the production of EVs by phytopathogens and their association with secondary metabolite cargo is available.

Here, we report the phytotoxic activity of indole alkaloids and EVs produced by *P. digitatum* in germination assays of Citrus sinensis seeds. The export of tryptoquialanines in *P. digitatum* involved EVs. Untargeted metabolomics was applied to confirm the EV-mediated metabolite export in the possible hosts (citrus and stone fruits) affected by *P. digitatum*.

## RESULTS

### Phytotoxicity activity of tryptoquialanine.

**FIG 1:**
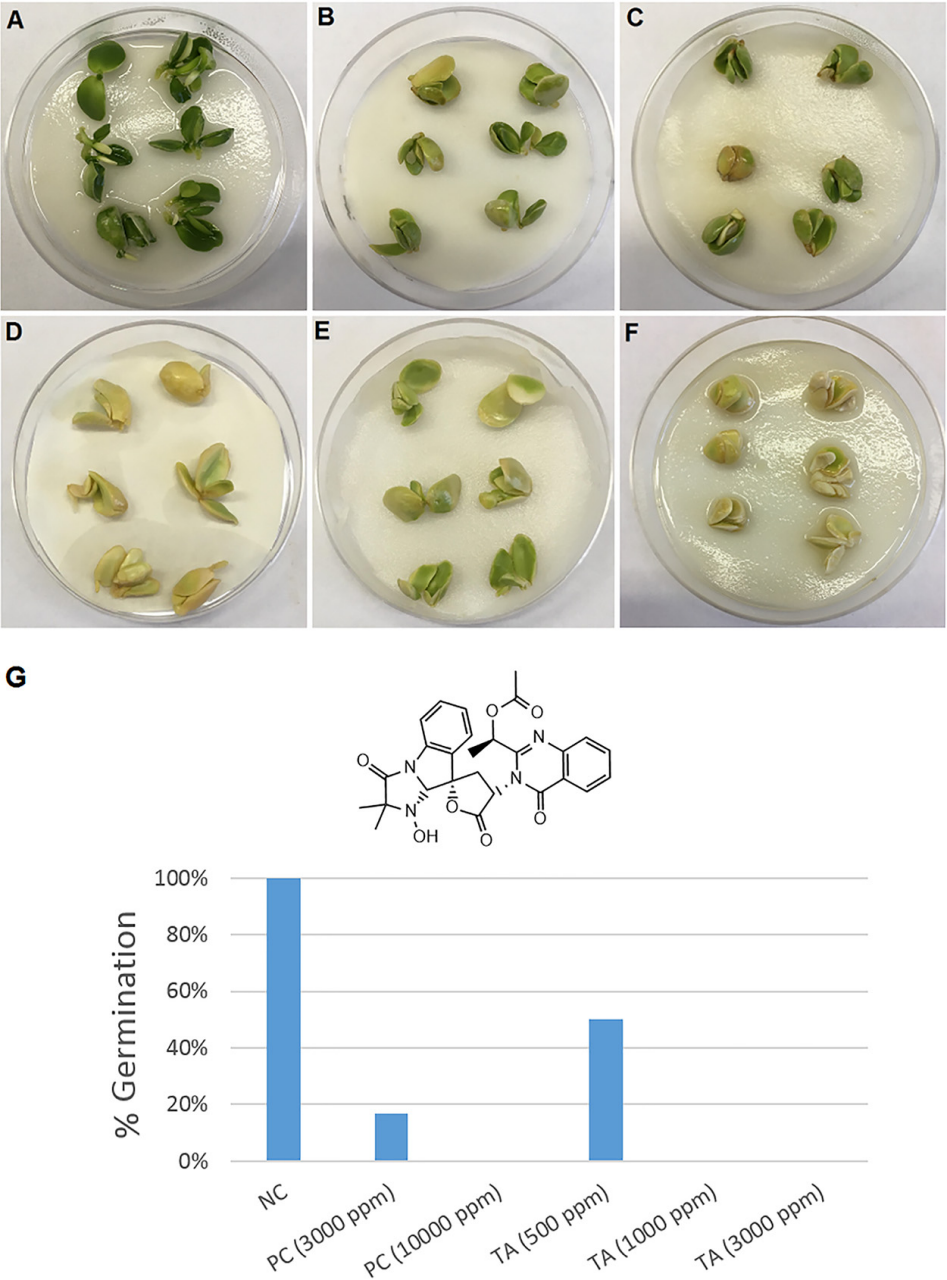
Tryptoquialanine A (TA) is an efficient inhibitor of germination in C. sinensis seeds. (A) Untreated seeds (negative control) showed a regular pattern of germination. (B to F) Seeds exposed to TA (500, 1,000, or 3,000 ppm; panels B, C, and D, respectively) or to the commercial herbicide Roundup (3,000 or 10,000 ppm; panels E and F, respectively) manifested defective germination. Seeds exposed to TA (3,000 ppm) and the positive control (PC) (10,000 ppm) did not germinate. (G) This visual perception was confirmed by the quantitative determination of germination (%) of C. sinensis seeds under different treatments. Six seeds were used in each treatment.

To investigate the pathogenic potential of tryptoquialanine A (TA), we first purified this alkaloid from *P. digitatum*’s crude extracts (Fig. S1). We then evaluated its phytotoxicity in germination assays of *Citrus sinensis* seeds. TA significantly inhibited seed germination at all tested concentrations. Seeds exposed to concentrations of TA lower than 1,000 ppm exhibited a delay in their germination time compared to that of the negative control (NC), as evidenced by the changes in seed color and size (Fig. 1). Seeds treated with the highest concentration of TA (3,000 ppm) showed a stronger phytotoxic effect, and no formation of radicle was observed (Fig. 1).

10.1128/mBio.03393-20.1 FIG S1 (A and B) HPLC chromatograms (280 nm) for (A) *P. digitatum* extract and (B) purified tryptoquialanine A. (C and D) HPLC-MS analysis of purified tryptoquialanine A (*m/z* 519); (C) extracted ion chromatogram and (D) mass spectrum. Download FIG S1, TIF file, 0.2 MB. Copyright © 2021 Costa et al. 2021 Costa et al. This content is distributed under the terms of the Creative Commons Attribution 4.0 International license.

**FIG 2:**
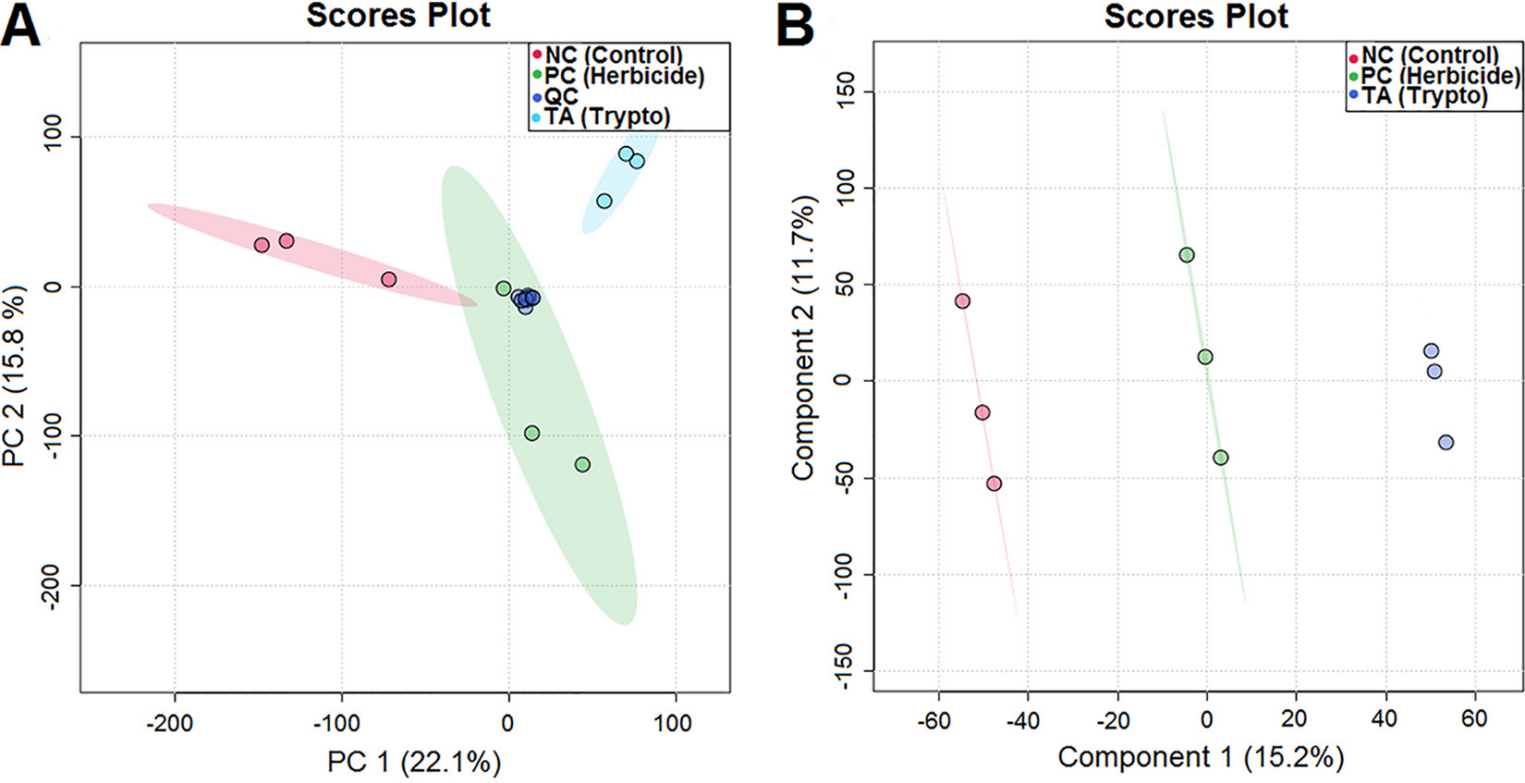
(A and B) PCA (A) and PLS-DA (B) for extracts of C. sinensis seeds treated with water (negative control), glyphosate (herbicide), or tryptoquialanine A (TA).

Following the observation of the phytotoxic activity of TA against the C. sinensis seeds, we compared the metabolite profiles of the seeds exposed to the different treatments by ultra-high-pressure liquid chromatography-mass spectrometry (UHPLC-MS). Principal-component analysis (PCA) of quality control (QC), negative control (NC), herbicide (PC), and tryptoquialanine A (TA) extracts was performed to observe data reproducibility and grouping tendencies (Fig. 2A). Data reproducibility was verified, as QC samples formed a distinct cluster. The two principal components, PC1 and PC2, were responsible for 37.9% of the variance of the data, revealing a separation between the seed groups related to the treatment received (water, glyphosate herbicide, or TA).

In order to verify the classification of the seeds according to the treatment received, partial least-squares discriminant analysis (PLS-DA) was performed. The PLS-DA score plot confirmed a clear separation between the seed groups (Fig. 2B). In PLS-DA, PC1 and PC2 accounted for 26.9% of the variance (15.2% for PC1 and 11.7% for PC2). As in the PCA score plot, control seeds were distributed in the opposite way of seeds treated with TA along PC1, while seeds treated with herbicide were plotted in the center.

### *P. digitatum* produces EVs *in vitro* and *in vivo*.

**FIG 3:**
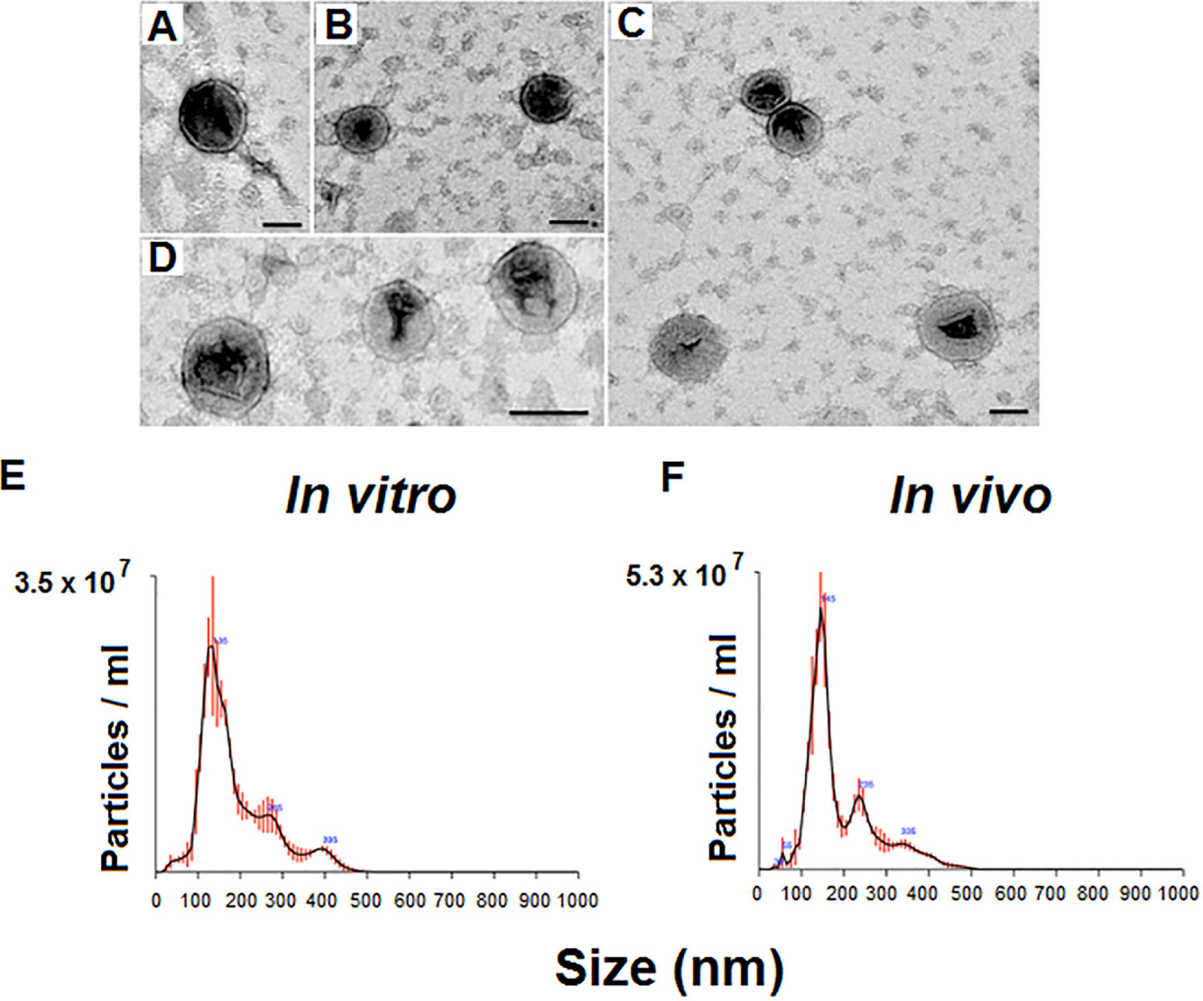
Production of EVs by *P. digitatum*. (A to D) Analysis of ultracentrifugation pellets by TEM revealed the presence of the typical round-shaped structures presenting double membranes. Similar results were obtained with samples obtained *in vivo* (panels A and B) and *in vitro* (panels C and D). The visual observations using TEM were confirmed using NTA, which detected particles mostly concentrated in the 100 to 200 nm range, with subpopulations in the 200 to 300 and 300 to 400 nm size ranges. (E and F) Similar results were obtained with *in vitro* (E) and *in vivo* (F) samples. One representative experiment of three independent replicates producing similar results is illustrated.

To inhibit the germination of C. sinensis seeds, TA is required to reach the extracellular environment. We then asked if the extracellular export of TA could be vesicle-mediated. However, the production of EVs by *P. digitatum* has not been reported so far. To address this question, we used methods for EV detection in different models of *P. digitatum* growth. Specifically, the production of EVs was evaluated in both solid agar medium and infected citrus fruits. Transmission electron microscopy (TEM) of *P. digitatum* samples grown *in vitro* revealed membranous structures with the typical features of vesicles, including round-shaped structures with bilayered membranes in the 100- nm size range (Fig. 3A to D). Similar results were observed for vesicles isolated from infected fruits. These results were confirmed by a second experimental approach. Nanoparticle tracking analysis (NTA) of the same samples revealed particles mostly concentrated in the 100- to 200-nm range, with subpopulations in the 200- to 300-nm and 300- to 400-nm size ranges (Fig. 3E and F). *In vitro* and *in vivo* samples had similar properties, which were consistent with those previously described for fungal EVs (19, 25, 26).

### Tryptoquialanine A is a component of *P. digitatum* EVs.

**FIG 4:**
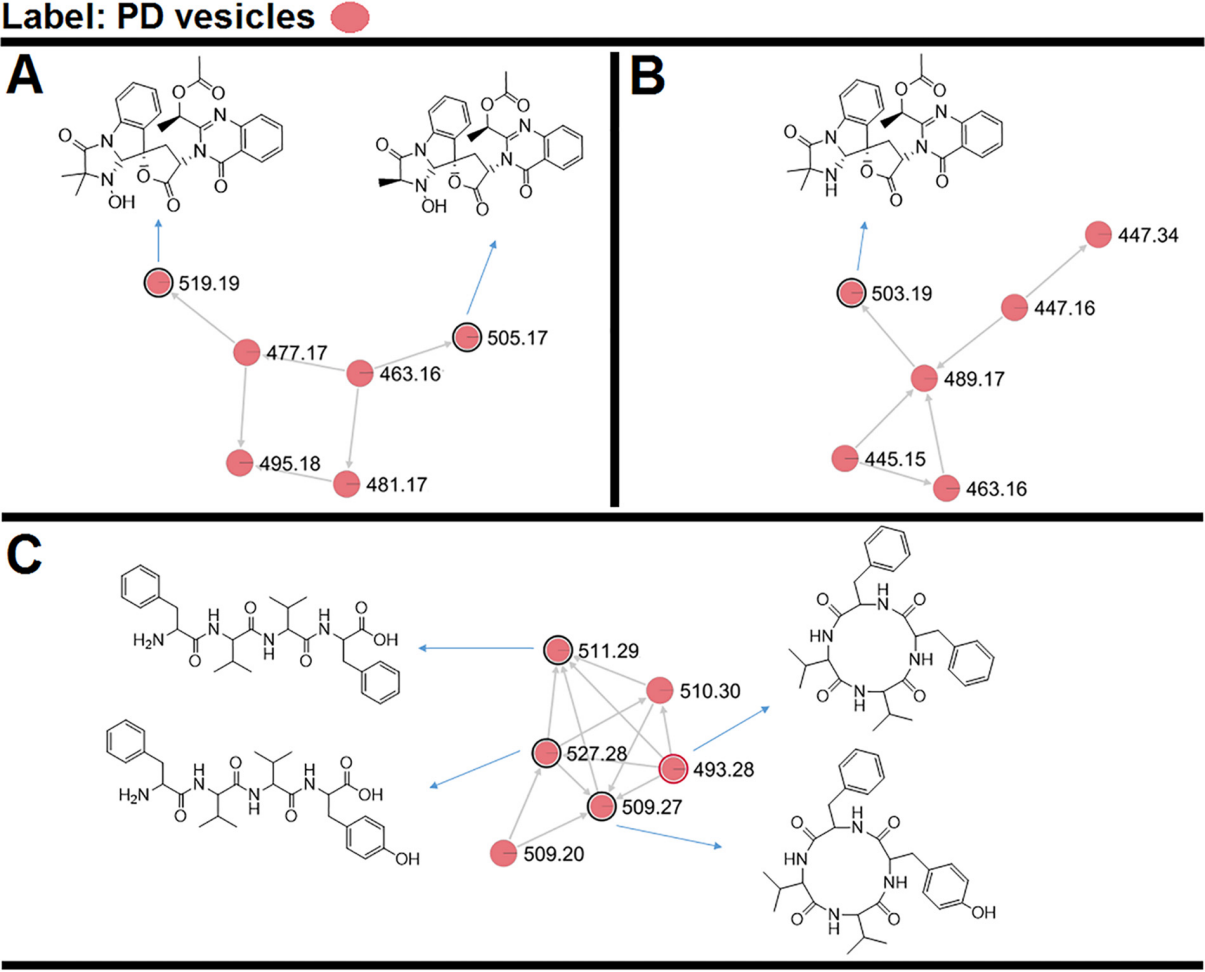
Molecular networking obtained for the *P. digitatum* EV cargo. (A to C) Indole alkaloids were grouped in clusters A and B, and tetrapeptides were grouped in cluster C. Nodes circled in black indicate molecules identified manually through exact masses and MS/MS fragmentation pattern comparison to the literature. Nodes circled in red indicate molecules identified by comparison with the GNPS platform database.

The metabolite composition of the *P. digitatum* EVs was investigated by UHPLC-MS/MS in EV extracts obtained *in vivo* (Fig. S2 and S3), followed by molecular networking in the Global Natural Products Social Molecular Networking (GNPS) platform and, when available, compared with standard metabolites. Molecular networking revealed three clusters (A, B, and C) that exhibited compounds present in the EVs (Fig. 4, pink symbols). Metabolites were manually identified by accurate mass analysis, MS/MS fragmentation profiles, or comparison with authentic standards (tryptoquialanine A and B) or as a hit in the GNPS database. The observed signals corresponded, respectively, to tryptoquialanine A (*m/z* 519.19), tryptoquialanine B (*m/z* 505.17), deoxytryptoquialanine (*m/z* 503.19), *cyclo*-(Phe-Val-Val-Tyr) (*m/z* 509.27), Phe-Val-Val-Phe (*m/z* 511.29), Phe-Val-Val-Tyr (*m/z* 527.28), and *cyclo*-(Phe-Phe-Val-Val) (*m/z* 493.28).

10.1128/mBio.03393-20.2 FIG S2 (A to C) Extracted ion chromatograms of *m/z* 519.18 for (A) *P. digitatum* EVs extract, (B) MS analysis control, and (C) EV isolation methodology control. (D) Mass spectrum of ion [M + H]^+^ *m/z* 519.1877 obtained for tryptoquialanine A (error, 0.61 ppm) at 9.2 min. (E to G) Extracted ion chromatograms of *m/z* 505.17 for (E) *P. digitatum* EV extract, (F) MS analysis control, and (G) EV isolation methodology control. (H) Mass spectrum of ion [M + H]^+^ *m/z* 505.1719 obtained for tryptoquialanine B (error, 0.26 ppm) at 8.7 min. (I to K) Extracted ion chromatograms of *m/z* 503.19 for (I) *P. digitatum* EV extract, (J) MS analysis control, and (K) EV isolation methodology control. (L) Mass spectrum of ion [M + H]^+^ *m/z* 505.1926 obtained for deoxytryptoquialanine (error = 0.23 ppm) at 8.6 min. Download FIG S2, TIF file, 0.2 MB. Copyright © 2021 Costa et al. 2021 Costa et al. This content is distributed under the terms of the Creative Commons Attribution 4.0 International license.

10.1128/mBio.03393-20.3 FIG S3 (A to C) Extracted ion chromatograms of *m/z* 509.27 for (A) *P. digitatum* EV extract, (B) MS analysis control, and (C) EV isolation methodology control. (D) Mass spectrum of ion [M + H]^+^ *m/z* 509.2761 obtained for *cyclo*-(Phe-Val-Val-Tyr) (error, 0.48 ppm) at 8.6 min. (E to G) Extracted ion chromatograms of *m/z* 511.29 for (E) *P. digitatum* EV extract, (F) MS analysis control, and (G) EV isolation methodology control. (H) Mass spectrum of ion [M + H]^+^ *m/z* 511.2917 obtained for Phe-Val-Val-Phe (error, 0.43 ppm) at 7.1 min. (I to K) Extracted ion chromatograms of *m/z* 527.28 for (I) *P. digitatum* EV extract, (J) MS analysis control, and (K) EV isolation methodology control. (L) Mass spectrum of ion [M + H]^+^ *m/z* 527.2866 obtained for Phe-Val-Val-Tyr (error, 0.40 ppm) at 6.4 min. (M to O) Extracted ion chromatograms of *m/z* 493.28 for (M) *P. digitatum* EV extract, (N) MS analysis control, and (O) EV isolation methodology control. (P) Mass spectrum of ion [M + H]^+^ *m/z* 493.2809 obtained for *cyclo*-(Phe-Phe-Val-Val) (error,= 0.03 ppm) at 9.7 min. Download FIG S3, TIF file, 0.3 MB. Copyright © 2021 Costa et al. 2021 Costa et al. This content is distributed under the terms of the Creative Commons Attribution 4.0 International license.

In the molecular networking analysis, each consensus MS/MS spectrum is represented by a node, and all nodes are labeled with their precursor mass. Indole alkaloids produced by *P. digitatum* were grouped in clusters A and B since they showed similar fragmentation patterns, with typical indole alkaloid fragments observed at [M+H]^+^ *m/z* 156.07, *m/z* 197.10, and *m/z* 213.10 (Fig. S4). Tryptoquialanines A and B and deoxytryptoquialanine are the final products of the tryptoquialanine biosynthetic pathway (27), and as already mentioned in this section, these indole alkaloids were reported as major secondary metabolites for *P. digitatum* (16). In cluster C, the GNPS database indicated the presence of *cyclo*-(Phe-Phe-Val-Val) (Fig. S5A), a mycotoxin known as fungisporin. We also observed that fungisporin analogues were grouped in this cluster. A fragmentation pattern with typical ions observed at [M+H]^+^ *m/z* 120.08, *m/z* 219.15, and *m/z* 247.14 was previously described for compounds Phe-Val-Val-Phe and Phe-Val-Val-Tyr (18, 28, 29) (Fig. S5B and C).

10.1128/mBio.03393-20.4 FIG S4 (A to F) Comparison of MS/MS spectra between (A) *P. digitatum* EV extract and (B) purified tryptoquialanine A, (C) *P. digitatum* EV extract and (D) purified tryptoquialanine B, and (E) *P. digitatum* EV extract and (F) purified deoxytryptoquialanine. Download FIG S4, TIF file, 0.1 MB. Copyright © 2021 Costa et al. 2021 Costa et al. This content is distributed under the terms of the Creative Commons Attribution 4.0 International license.

10.1128/mBio.03393-20.5 FIG S5 (A) MS/MS match between GNPS database (green) and compound *cyclo*-(Phe-Phe-Val-Val) (black). (B and C) MS/MS spectrum of (B) *m/z* 511.29 and (C) *m/z* 527.28 obtained from *P. digitatum* EV extract. The fragmentation pattern was compared with MS/MS data of compounds Phe-Val-Val-Phe and Phe-Val-Val-Tyr reported in the literature. Download FIG S5, TIF file, 0.08 MB. Copyright © 2021 Costa et al. 2021 Costa et al. This content is distributed under the terms of the Creative Commons Attribution 4.0 International license.

### Quantification of tryptoquialanine A in *P. digitatum* EVs.

The quantitative composition of alkaloids in *P. digitatum* EVs was evaluated using UHPLC-MS/MS analyses. First, a calibration curve was prepared using standard TA (t_R_ = 7.2 min) (Fig. S1) purified from *P. digitatum*’s crude extracts (17). The coefficient of determination (r^2^) obtained was greater than 0.998, indicating an excellent linearity (Fig. S6). Extracts of *P. digitatum* EVs isolated from *in vivo* assays were again analyzed for the presence of TA. Each 1.0 × 10^10^ *P. digitatum* EV contained 0.0184 ± 0.0002 μg of TA.

10.1128/mBio.03393-20.6 FIG S6 UHPLC-MS/MS calibration curve obtained for tryptoquialanine A. Download FIG S6, TIF file, 0.01 MB. Copyright © 2021 Costa et al. 2021 Costa et al. This content is distributed under the terms of the Creative Commons Attribution 4.0 International license.

### *P. digitatum* EVs are phytotoxic to seeds.

We asked whether the phytotoxic effects of TA alone would be comparable to its vesicle-exported form. To address this question, we isolated EVs produced during infection and performed the seed germination tests in the presence of the vesicles. EVs were adjusted to a final concentration of 2.1 × 10^10^ EVs ml^−1^ to allow comparisons between the effects of purified TA and the vesicle preparations.

**FIG 5:**
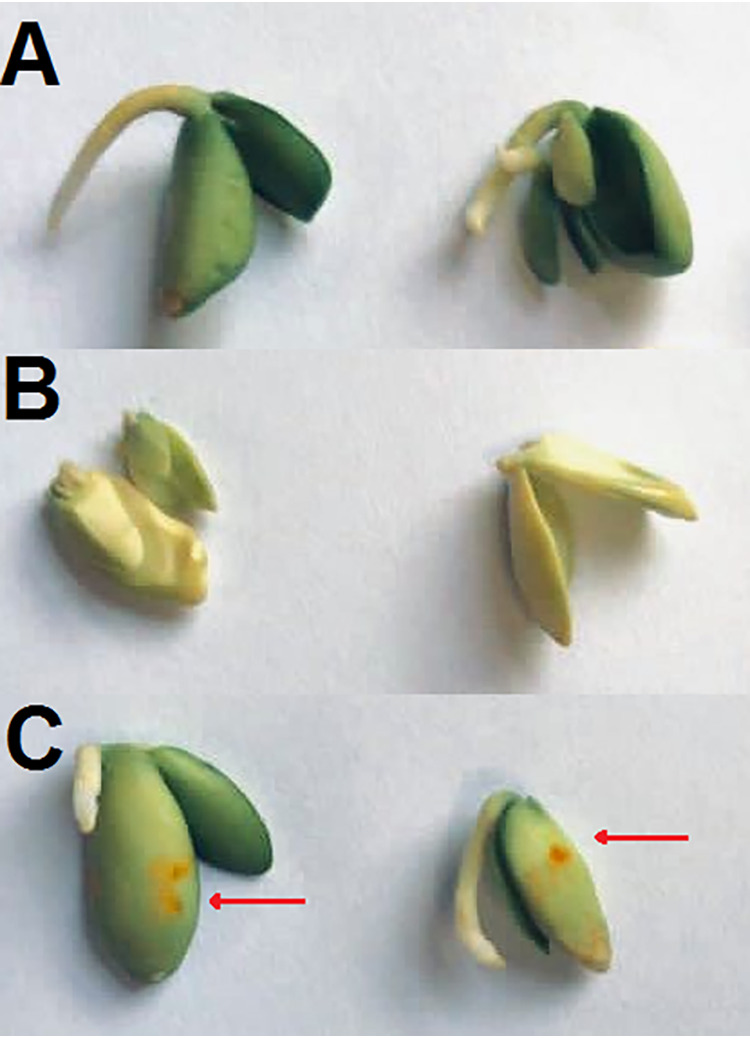
*P. digitatum* EVs affect C. sinensis seeds. (A) Negative control (NC), consisting of C. sinensis seeds incubated in PBS. (B) Positive control (PC), consisting of the citrus seeds incubated in glyphosate (10,000 ppm). (C) Incubation of C. sinensis seeds with *P. digitatum* EVs produced *in vivo* (2.1 × 10^10^ EVs ml^−1^). Seeds exposed to fungal vesicles presented injured tissues (orange spots on their surface; red arrows).

After 10 days of incubation, seeds exposed to EVs had germination rates similar to those observed in untreated systems. Positive controls of inhibition of germination revealed seeds with different colors and patterns and absence of radicle formation, as expected. However, the seeds that were exposed to the *P. digitatum* EVs showed altered tissues. Tissular alteration included injured areas with differences in pigmentation (Fig. 5). No color alteration or tissue damage was observed in the negative controls.

### Comparison of secondary metabolite production of *P. digitatum* in different hosts.

**FIG 6:**
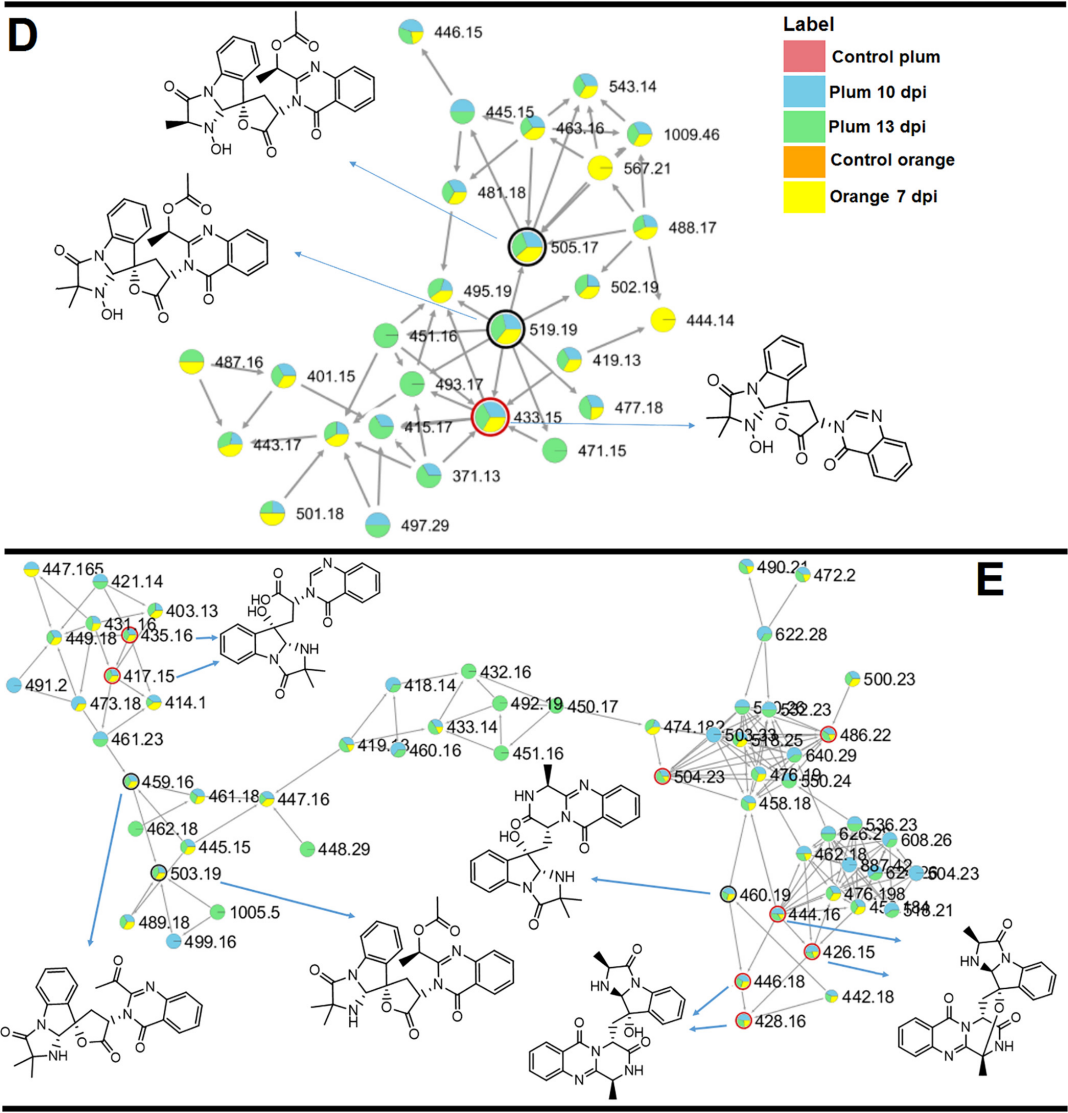
MS/MS molecular network of different extracts of *P. digitatum.* Nodes circled in black indicate molecules identified manually through accurate mass and fragmentation pattern analysis. Nodes circled in red indicate molecules identified by comparison with the GNPS database. Indole alkaloids produced by *P. digitatum* were grouped in clusters D and E.

**FIG 7:**
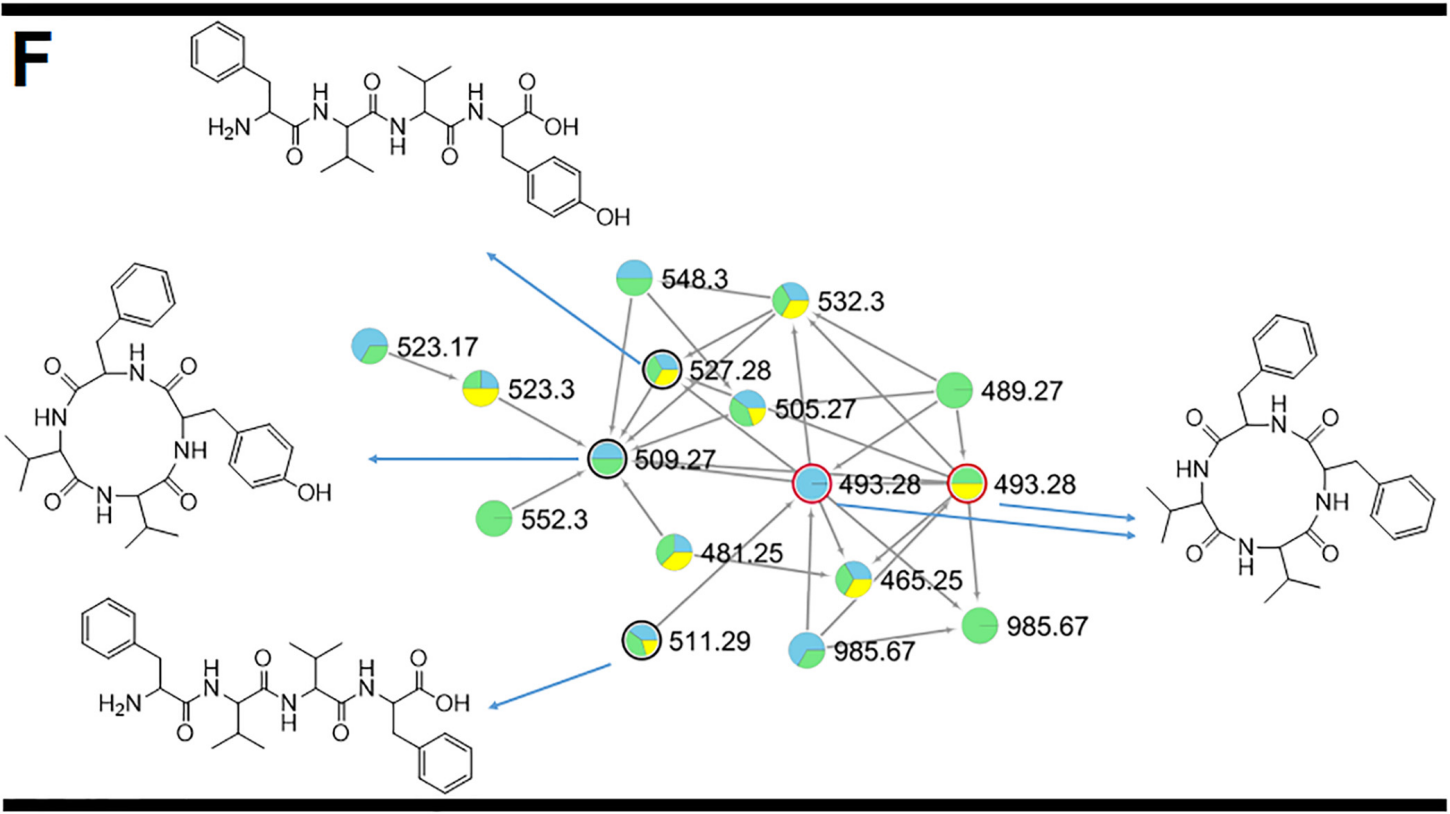
MS/MS molecular network of different extracts of *P. digitatum.* Nodes circled in black indicate molecules identified manually through accurate mass and fragmentation pattern analysis. Nodes circled in red indicate molecules identified by comparison with the GNPS database. Fungisporin and analogues were grouped in cluster F.

**FIG 8:**
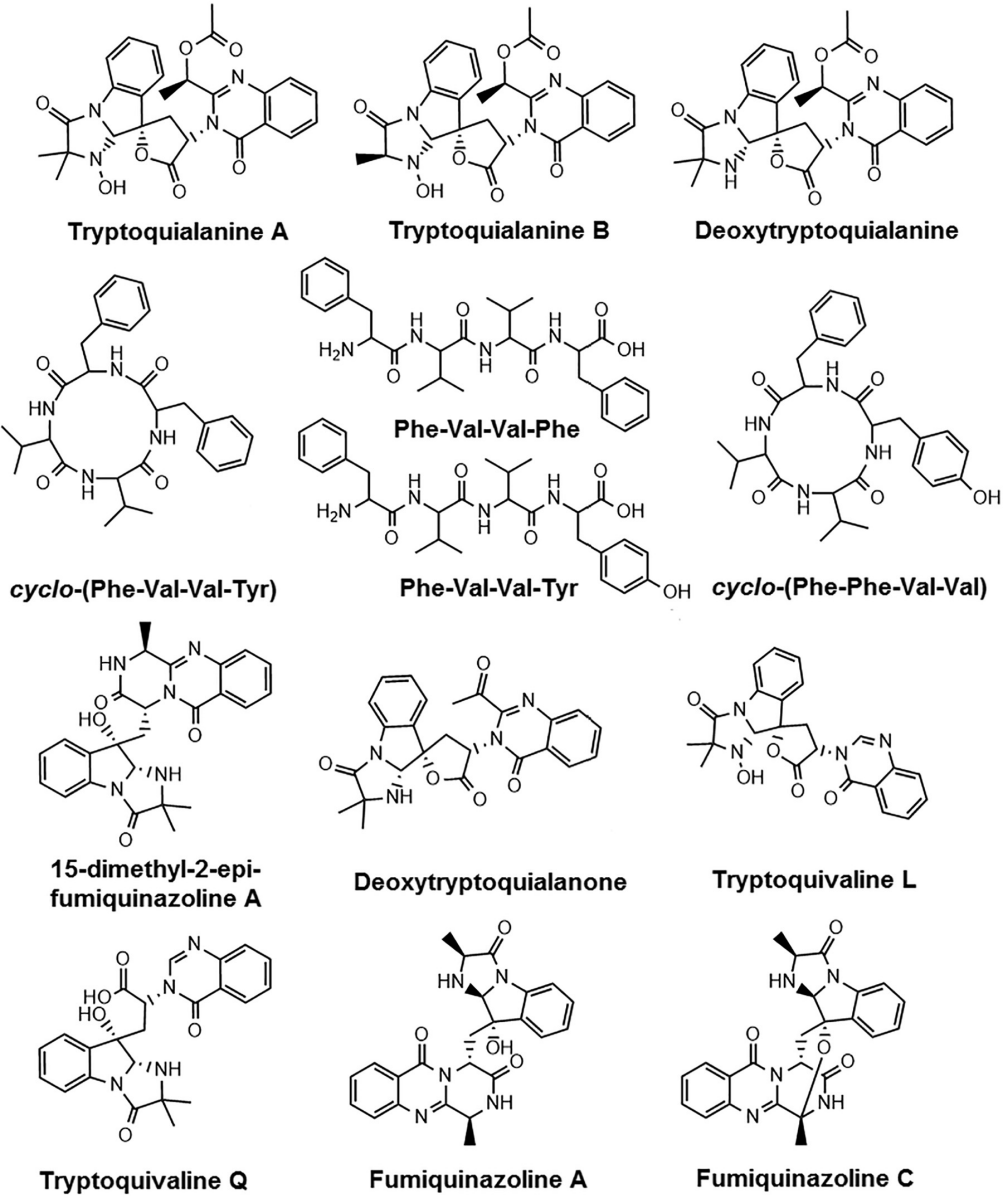
Structures of secondary metabolites identified manually or through the GNPS MS/MS database.

After evaluating the citrus response to TA and EVs, we next analyzed the metabolite response of different hosts to the infection caused by *P. digitatum*. Similar to what we observed for the *P. digitatum* EV extracts, molecular networking of extracts from plums and oranges infected with *P. digitatum* showed three clusters (D, E, and F) with compounds present only in the infected fruits (blue, green, and yellow nodes) and absent in control fruits (orange and pink nodes) (Fig. 6 and 7). Metabolites were manually identified by their accurate masses and fragmentation profiles or identified as hits in the GNPS database. Fragmentation patterns obtained by MS/MS analyses are represented in Fig. S7. Accurate mass measurements showed mass errors below 5 ppm (Table 1). The structures of the detected metabolites are shown in Fig. 8.

**TABLE 1:** MS data obtained for *P. digitatum* secondary metabolites observed on GNPS molecular network

Compound	Ion formula ([M + H]^+^)	Calculated *m/z*	Exptl *m/z*	Error (ppm)
Tryptoquialanine A	C_27_H_27_N_4_O_7_	519.1874	519.1876	–0.4
Tryptoquialanine B	C_26_H_25_N_4_O_7_	505.1723	505.1735	2.4
Deoxytryptoquialanine	C_27_H_27_N_4_O_6_	503.1925	503.1948	4.6
15-dimethyl-2-epi-fumiquinazoline A	C_25_H_26_N_5_O_4_	460.1979	460.1983	0.9
Deoxytryptoquialanone	C_25_H_23_N_4_O_5_	459.1663	459.1678	3.3
Tryptoquivaline L	C_23_H_21_N_4_O_5_	433.1512	433.1523	2.5
Tryptoquivaline Q	C_23_H_23_N_4_O_5_	435.1668	435.1673	1.1
Fumiquinazoline A	C_24_H_24_N_5_O_4_	446.1828	446.1838	2.2
Fumiquinazoline C	C_24_H_22_N_5_O_4_	444.1672	444.1687	3.4
*cyclo*-(Phe-Phe-Val-Val)	C_28_H_37_N_4_O_4_	493.2814	493.2822	1.6
*cyclo*-(Phe-Val-Val-Tyr)	C_28_H_37_N_4_O_5_	509.2763	509.2770	1.4
Phe-Val-Val-Phe	C_28_H_39_N_4_O_5_	511.2920	511.2920	0.0
Phe-Val-Val-Tyr	C_28_H_39_N_4_O_6_	527.2869	527.2859	–1.9

10.1128/mBio.03393-20.7 FIG S7 (A to M) Mass spectrum and MS/MS spectrum for (A) tryptoquialanine A ion [M + H]^+^ *m/z* 519.1876, (B) tryptoquialanine B ion [M + H]^+^ *m/z* 505.1735, (C) deoxytryptoquialanine ion [M + H]^+^ *m/z* 503.1948, (D) *cyclo*-(Phe-Val-Val-Tyr) ion [M + H]^+^ *m/z* 509.2770, (E) Phe-Val-Val-Phe ion [M + H]^+^ *m/z* 511.2920, (F) Phe-Val-Val-Tyr ion [M + H]^+^ *m/z* 527.2859, (G) *cyclo*-(Phe-Phe-Val-Val) ion [M + H]^+^ *m/z* 493.2822, (H) 15-dimethyl-2-epi-fumiquinazoline A ion [M + H]^+^ *m/z* 460.1983, (I) deoxytryptoquialanone ion [M + H]^+^ *m/z* 459.1678, (J) tryptoquivaline L ion [M + H]^+^ *m/z* 433.1523, (K) tryptoquivaline Q ion [M + H]^+^ *m/z* 435.1673, (L) fumiquinazoline A ion [M + H]^+^ *m/z* 446.1838, and (M) fumiquinazoline C ion [M + H]^+^ *m/z* 444.1687. Download FIG S7, TIF file, 1.1 MB. Copyright © 2021 Costa et al. 2021 Costa et al. This content is distributed under the terms of the Creative Commons Attribution 4.0 International license.

The compounds identified in our analysis included 15-dimethyl-2-*epi*-fumiquinazoline (*m/z* 460.19) A, deoxytryptoquialanone (*m/z* 459.16), tryptoquivaline L (*m/z* 433.15), tryptoquivaline Q (*m/z* 435.16 and 417.15), fumiquinazoline A (*m/z* 446.18 and 428.16), and fumiquinazoline C (*m/z* 444.16 and 426.15). Compounds 15-dimethyl-2-*epi*-fumiquinazoline A and deoxytryptoquialanone are intermediates of the tryptoquialanine biosynthetic pathway (27), while the tryptoquivalines and fumiquinazolines were previously identified as *P. digitatum* metabolites (17). Fungisporin and analogues were also identified in EVs (cluster C, Fig. 4). A few differences were observed in clusters D, E, and F (Fig. 7 and 8) considering the production of secondary metabolites by *P. digitatum* in different fruits. All identified compounds were detected in infected plums (at 10 and 13 days postinoculation[dpi]) and oranges (at 7 dpi) (Fig. S8).

10.1128/mBio.03393-20.8 FIG S8 Fruits infected with *P. digitatum.* (A to C) (A) plum at 10 dpi, (B) plum at 13 dpi, and (C) orange at 7 dpi. Download FIG S8, TIF file, 0.3 MB. Copyright © 2021 Costa et al. 2021 Costa et al. This content is distributed under the terms of the Creative Commons Attribution 4.0 International license.

The complete molecular networking obtained for *P. digitatum* is represented in the supplemental information (Fig. S9). *P. digitatum* molecular networking was composed of 235 clusters, 83 of which (36%) were composed of unknown metabolites that were only present in the infected fruits and absent in the control fruits. Molecular networking also showed clusters containing unknown metabolites present only in infected oranges or only in infected plums (Fig. S10).

10.1128/mBio.03393-20.9 FIG S9 Complete molecular networking obtained for extracts of plums and citrus fruits infected by *P. digitatum*. Download FIG S9, JPG file, 2.7 MB. Copyright © 2021 Costa et al. 2021 Costa et al. This content is distributed under the terms of the Creative Commons Attribution 4.0 International license.

10.1128/mBio.03393-20.10 FIG S10 Examples of clusters obtained by molecular networking for extracts of *P. digitatum* infection in different fruits. The unknown compounds are present in just one type of the fruits and absent in the control fruits Download FIG S10, TIF file, 0.03 MB. Copyright © 2021 Costa et al. 2021 Costa et al. This content is distributed under the terms of the Creative Commons Attribution 4.0 International license.

## DISCUSSION

Tryptoquialanines are the major secondary metabolites produced by *P. digitatum* (16). The involvement of tryptoquialanines during the infection of citrus fruits by *P. digitatum* was evaluated after deletion of the *tqaA* gene (nonribosomal peptide synthetase) responsible for the biosynthesis of tryptoquialanines. *P. digitatum* mutants deficient in tryptoquialanine A production did not have their virulence affected compared to wild-type *P. digitatum* cells (30). Thus, tryptoquialanines were initially thought to be dispensable for the pathogenesis in fruits. Other damaging roles could not be ruled out, since they were not investigated in detail. It has been recently reported that TA is accumulated in the citrus surface during the *P. digitatum* pathogenic process (17), suggesting extracellular export. TA also exhibited insecticidal activity against Aedes aegypti larvae (17). These results suggested that tryptoquialanines are involved in the fruit protection against insects that could compete with the fungus for the rotten fruit (17). In coculture models, it has been observed that *P. digitatum* tryptoquialanines were present in the confrontation zone with citrus pathogens, suggesting that tryptoquialanines participate in antifungal defense mechanisms that could provide competitive advantages during infection of the citrus host (18).

The reports described above and the fact that tryptoquialanines are the major metabolites produced by *P. digitatum* led us to ask if these metabolites could be involved in *P. digitatum* phytotoxic activity. To address this question, we investigated the phytotoxic effects of tryptoquialanines in a seed germination model, as previously established for the evaluation of the phytotoxicity of chemicals (31, 32). This method is simple, sensitive, and of low cost (31). Seed germination is a vulnerable stage in the plant life cycle, during which seedlings are weak, sensitive, and more affected by unfavorable conditions (32). Our results indicated that TA was comparable to the herbicide Roundup in its ability to inhibit germination. Such an inhibitory effect requires the extracellular export of TA, as suggested by its accumulation on the surface of citrus infected with *P. digitatum* (17). We then hypothesized that the transport of the indole alkaloids from *P. digitatum* cells to the extracellular environment would involve EVs, as previously described for fungal proteins, glycans, and RNA (25, 33). In our model, EVs were detected in culture and infected citrus fruits. The possibility of coisolation of plant EVs in the *in vivo* samples cannot be ruled out, since it is well known that plant cells also produce EVs during interaction with fungi (34). However, the similar features of EVs obtained *in vivo* and *in vitro* and our vesicle compositional analysis reinforce the notion that *P. digitatum* produces EVs *in vitro* and during plant infection. The observation of *P. digitatum* EVs gains additional significance considering that most of the studies characterizing fungal EVs used human pathogens as models, which implies that the importance of EV production by phytopathogens has been underscored so far. In this context, it has been only recently demonstrated that EVs from the cotton pathogen Fusarium oxysporum f. sp. *vasinfectum* induce a phytotoxic response in plants (25). In the EV cargo of F. oxysporum f. sp. v*asinfectum*, 482 enzymes were identified, including two polyketide synthases, yet the isolated EVs presented a deep purple color, indicating that a naphthoquinone pigment is packaged into the EVs (25). The authors suggested that EVs could be a site of biosynthesis and transport of pigments and other secondary metabolites (25), an idea that is quite complementary to what is presented in our study.

Secondary metabolites participate in the virulence mechanisms of some phytopathogenic fungi, implying that knowledge of metabolite exportation could improve the understanding of the molecular basis of plant infection and fruit protection (4, 15). However, the association of fungal metabolites and EVs has not been established so far in plant infection models. Based on the observation of *P. digitatum* EVs *in vitro* (potato dextrose agar) and *in vivo* (citrus fruits), we identified indole alkaloids and mycotoxins in EV samples. *Penicillium* species are known to produce mycotoxins such as fungisporin (35, 36). Fungisporin and analogues were reported in the cultures of P. canescens (28), P. roqueforti (29), P. citrinum (18) and P. chrysogenum (37). Therefore, the production of fungisporin compounds by *P. digitatum* was expected. To the best of our knowledge, the presence of secondary metabolites and mycotoxins in EVs produced by a phytopathogen *in vivo* is reported here for the first time.

An estimate of the tryptoquialanine levels in EVs could provide insights into the biosynthesis and metabolic flow of these molecules in *P. digitatum*. Previous studies with oranges infected by *P. digitatum* showed that, at 5 days postinfection, TA was detected in the orange epicarp, mesocarp, and endocarp, with concentrations of 24.810, 388, and 24 μg kg^−1^, respectively (38). TA concentration in the EVs was considerably lower. We then speculated that the biosynthesis of tryptoquialanines may occur in the fungal cells with further export in EVs. This mechanism would differ from that described by Bleackley et al. in the F. oxysporum f. sp. v*asinfectum* EVs (25).

*P. digitatum* EVs induced alterations in the C. sinensis seeds, as concluded from the observation of color alteration resulting from tissue lesions. Similar results were reported in recent studies with cotton cotyledons infiltrated with F. oxysporum f. sp. *vasinfectum*. In this model, EVs induced discoloration around the sites of infiltration (25). Therefore, the phytotoxic effect observed for the isolated TA was different from that caused by the EVs. These differences were, in fact, expected, considering that vesicular TA is accompanied by hundreds of other molecules. Those molecules could, for instance, physically interact with TA, altering its relative concentration. In addition, if those additional vesicular molecules have biological effects that differ from those observed for TA alone, it would be very hard to predict what kind of effect would prevail, since the relative concentration of vesicular molecules in the *P. digitatum* model is still unknown. In any case, our results provide a proof-of-concept model showing that *P. digitatum* exports bioactive molecules in EVs that can directly impact the pathogenic process.

*P. digitatum* pathogenesis was believed to be restricted to citrus fruits (13). However, this fungus is also an aggressive pathogen of stone fruits, including nectarines and plums (39, 40). Few studies have investigated the infection of stone fruits by *P. digitatum*. Even though *P. digitatum* disease was characterized at the physical (incidence, lesion diameter, pH) and molecular (gene expression) levels (40, 41), no information on secondary metabolite production has been presented in the literature for this host-pathogen interaction. Since tryptoquialanines and mycotoxins were found in EVs produced during infection, the metabolic profile of *P. digitatum* in different fruits was evaluated in order to verify if the same metabolites were found in the different hosts. Molecular networking analyses indicate that intermediates of the tryptoquialanine biosynthetic pathway are present in fruits and absent in EVs. These data are in agreement with the quantification level of TA in EVs, reinforcing the idea that tryptoquialanines are only transported by the EVs. Also, our results are the first to identify the production of tryptoquialanines and other indole alkaloids in the *P. digitatum*-stone fruit interaction. Likewise, the similarity between the metabolic profile in the fruits suggests that the production of EVs by *P. digitatum* is not restricted to the citrus fruits, since the same metabolites found in EV cargo obtained from infection in citrus were detected in plums. The clusters containing unknown metabolites present only in infected oranges or only in infected plums (Fig. S9) suggest that the metabolite production of *P. digitatum* can vary depending on the infected fruit.

### Conclusions.

This work is the first to report that *P. digitatum* is able to release EVs and to report secondary metabolites in EVs produced by a phytopathogen *in vivo.* Furthermore, we suggested that TA is synthesized intracellularly and exported in EVs. Molecular networking confirmed our hypothesis that tryptoquialanines and mycotoxins are delivered through EVs during the infection process, since the intermediates of the tryptoquialanine biosynthetic pathway are absent in the EVs. This delivery system is not restricted to citrus and occurs in different types of fruits, such as plums.

A novel phytotoxic function for *P. digitatum* EVs and for tryptoquialanines was observed. EVs caused alterations in the physiology of C. sinensis seed tissues, while TA inhibited 100% of seed germination. The presence of alkaloids and mycotoxins in phytotoxic EVs opens new venues for the investigation of fungal secretion and its relationship with plant pathogenesis. Also, our results provided new insights into the biological role of the indole alkaloids and the infection strategies used by the phytopathogen *P. digitatum*.

## MATERIALS AND METHODS

### Fungal strain and culture conditions.

The *P. digitatum* strain is deposited in the Spanish Type Culture Collection (CECT) (accession code CECT20796). The fungus was cultured in commercial potato dextrose agar (PDA) (darkness, 7 days at 25°C). Conidial suspensions were prepared in sterile distilled water and adjusted to a final concentration of 1.0 × 10^6^ conidia ml^−1^.

### Purification of tryptoquialanine A by high-performance liquid chromatography (HPLC).

*P. digitatum* was cultivated in 12 liters of PDA distributed in petri dishes. After cultivation, the content of the petri dishes was sliced and transferred to Erlenmeyer flasks. The content of the Erlenmeyer flasks was extracted twice with ethyl acetate (EtOAc) under sonication in an ultrasonic bath for 1 h. The mixture of agar, mycelia, and EtOAc was filtered, and the solvent was removed under reduced pressure.

The *P. digitatum* EtOAc extract was suspended in methanol (MeOH), filtered, and subjected to separation by high-performance liquid chromatography (HPLC) in order to obtain pure tryptoquialanine A. HPLC separation was performed with a Phenomenex column Luna 5-μm phenyl-hexyl (250 × 4.6 mm) using a Shimadzu prominence HPLC LC-20AT instrument connected to a CBM-20A communication bus module, to an SPD-M20A photodiode array detector, and to a SIL-20A auto sampler. The mobile phases were 0.1% (vol/vol) formic acid in water (A) and acetonitrile (B). The flow rate was 1.0 ml min^−1^. Elution was performed as follows (A:B): gradient from 95:5 up to 55:45 for 30 min, then up to 35:65 from 30 to 52 min, then up to 5:95 from 52 to 55 min, remaining under this condition for 5 min. Column reconditioning between each injection was a gradient to 95:5 from 60 to 61 min, remaining under this condition for 9 min. Semipreparative HPLC separations were performed with a Phenomenex column Luna 5 μm phenyl-hexyl (250 × 10 mm) using a Waters 1525 binary HPLC pump equipped with a Waters 2998 photodiode array detector and a Waters fraction collector III. The eluent was the same as indicated above with a flow rate of 4.7 ml min^−1^.

### Seed germination test (phytotoxicity assay).

The phytotoxicity of tryptoquialanine A on seed germination was evaluated as previously described with a few modifications (42–45). Briefly, C. sinensis seeds were manually collected from oranges purchased at a local grocery store (Campinas, São Paulo, Brazil). Seeds coats were removed, and seeds were immersed in a 50% (vol/vol) commercial bleach solution for 15 min for surface sterilization. Six sterilized seeds were placed in each petri dish (6 cm) lined with two filter papers. A volume of 2.5 ml of treatment solution was added to the plate. As the negative control (NC), seeds were treated with sterile distilled H_2_O containing dimethyl sulfoxide (DMSO) 3% (vol/vol). Tryptoquialanine A (TA) was solubilized in DMSO and diluted in sterile distilled water to a final concentration of 500, 1,000, and 3,000 ppm. The commercial herbicide Roundup was utilized as a positive control (PC) diluted to the concentrations of 10,000 and 3,000 ppm in sterile distilled water containing DMSO 3% (vol/vol). Treatment solutions were filtered through 0.22-μm membranes. Petri dishes were sealed with tape and incubated in a biochemical oxygen demand (BOD) chamber at 25°C with photoperiods of 12 h for 10 days. After incubation, the percentage of seed germination was calculated as described in Equation 1, considering complete, proportionate, and healthy development.

(1) $$\text{\% }\mathrm{Germination}=\frac{\mathrm{number}\;\mathrm{of}\;\mathrm{germinated}\;\mathrm{seeds}}{\mathrm{total}\;\mathrm{number}\;\mathrm{of}\;\mathrm{seeds}\;}\times 100$$

To evaluate the phytotoxic activity of EVs, uncoated and sterilized C. sinensis seeds were placed in a 24-well cell culture plate lined with filter papers (1 seed per well). The seeds were treated with 100 μl of a phosphate-buffered saline (PBS) solution of *P. digitatum* EVs (2.1 × 10^10^ EVs ml^−1^). Negative controls (NC) were performed using 100 μl of PBS, and for positive controls (PC), 100 μl of the herbicide Roundup diluted in PBS (10,000 ppm) was used. The plate was sealed and incubated as described above.

### Infection of fruits by *P. digitatum* (*in vivo* assays) and metabolite extraction.

For *in vivo* assays, mature oranges (C. sinensis) and plums (Prunus salicina) obtained from a local grocery store (Campinas, São Paulo, Brazil) were surfaced sterilized and wounded (17). Four fruits (2 oranges and 2 plums) were infected with 15 μl of a *P. digitatum* 1.0 × 10^6^ conidia ml^−1^ solution. Control fruits (2 oranges and 2 plums) were also included. Infected and control fruits were stored in sterile 500-ml beakers in darkness at 25°C. The fruits were incubated for different numbers of days postinoculation (dpi) in triplicates.

After the infection period (7 dpi for oranges, 10 and 13 dpi for plums), extraction of infected fruits was performed as previously described, with few modifications (46). Fruits were cut around the infected area (4 cm by 4 cm), and collected fruit pieces were extracted with 5 ml of MeOH for 1 h in ultrasonic bath. The same procedure was performed for control fruits. MeOH extracts were filtered, dried with a N_2_ flux, and stored at −20°C.

### Isolation of *P. digitatum* EVs and metabolite extraction.

Isolation of *P. digitatum* EVs produced *in vitro* was performed as previously described, with a few modifications (26). Fungal cells were cultivated and softly scraped from PDA plates (triplicates, 20 ml of PDA per plate) using a sterile spatula. Fungal mycelia were transferred to a Falcon tube filled with 30 ml of sterile phosphate-buffered saline (PBS). For the analysis of EVs *in vivo*, nine oranges (C. sinensis) were infected with *P. digitatum* (as described above). Infected fruits were incubated for 7 days (darkness, 25°C). Then, fungal cells in the infected areas of fruits were softly scraped using a sterile spatula and transferred to a Falcon tube filled with 30 ml of PBS. Then, 30-ml cell suspensions obtained *in vivo* or *in vitro* were sequentially centrifuged to remove fungal cells (5,000 × *g* for 15 min at 4°C) and possible debris (15,000 × *g* for 15 min at 4°C). The remaining supernatants were filtered through 0.45-μm-pore syringe filters and ultracentrifuged to collect EVs (100,000 × *g* for 1 h at 4°C). Ultracentrifugation pellets were negatively stained and analyzed by transmission electron microscopy (TEM) as previously described (26). Briefly, EV samples were transferred to carbon- and Formvar-coated grids and negatively stained with 1 % (vol/vol) uranyl acetate for 10 min. The grids were then blotted dry before immediately being observed in a JEOL 1400Plus transmission electron microscope at 90 kV. The same samples were subjected to nanoparticle tracking analysis (NTA) on an LM10 nanoparticle analysis system, coupled with a 488-nm laser and equipped with an _S_CMOS camera and a syringe pump (Malvern Panalytical, Malvern, United Kingdom). Recorded data were acquired and analyzed using the NTA v.3.0 software (Malvern Panalytical).

To study the vesicular cargo, EVs obtained *in vivo* were extracted with 1 ml of MeOH HPLC grade for 1 h in an ultrasonic bath.

### Mass spectrometry (MS) analyses.

***In vivo* extracts.** *in vivo* extracts were resuspended in 1 ml of MeOH HPLC grade. An aliquot of 100 μl was diluted in 900 μl of MeOH HPLC grade, filtered through 0.22-μm membranes, and collected in glass vials. UHPLC-MS analyses were performed in a Waters Acquity UPLC H-class chromatograph coupled to a Waters Xevo G2-XS QToF mass spectrometer using electrospray ionization. The conditions were as follows: positive mode, capillary voltage at 1.2 kV; source temperature at 100°C; cone gas (N_2_) flow of 50 liters h^−1^; desolvation gas (N_2_) flow of 750 liters h^−1^, and *m/z* range of 100 to 1,500. MS/MS analyses were performed using a collision energy ramp of 6 to 9 V (low mass) and 60 to 80 V (high mass). A BEH C_18_ column (2.1 mm by 100 mm by 1.7 μm) was used. Mobile phases were 0.1% (vol/vol) formic acid in water (A) and acetonitrile (B). Eluent profile (A:B) 0 to 6 min, gradient from 90:10 up to 50:50; 6 to 9 min, gradient up to 2:98; 9 to 10 min, gradient up to 90:10. The flow rate was 0.2 ml min^−1^. The injection volume was 2 μl. Operation and spectrum analyses were conducted using Waters MassLynx v.4.1. software.

***P. digitatum* EV extracts.** First, 1 ml of EV extracts was filtered through 0.22-μm membranes into glass vials. UHPLC-MS analyses were performed using a Thermo Scientific QExactive hybrid Quadrupole-Orbitrap mass spectrometer with the following parameters: electrospray ionization in positive mode, capillary voltage at +3.5 kV; capillary temperature at 250°C; S-lens of 50 V, and *m/z* range of 133.40 to 2,000.00. MS/MS was performed using normalized collision energy (NCE) of 30 eV, and 5 precursors per cycle were selected. Stationary phase: Thermo Scientific Accucore C18 2.6 μm (2.1 mm x 100 mm) column. Mobile phases were 0.1% (vol/vol) formic acid in water (A) and acetonitrile (B). Eluent profile (A:B) 0 to 10 min, gradient from 95:5 up to 2:98; held for 5 min; 15 to 16.2 min gradient up to 95:5; held for 8.8 min. The flow rate was 0.2 ml min^−1^. The injection volume was 3 μl. Operation and spectrum analyses were conducted using Xcalibur software (v.3.0.63) developed by Thermo Fisher Scientific.

### Seed extracts.

Two seeds of each condition, TA (3,000 ppm), PC (10,000 ppm), and NC, were macerated with liquid nitrogen in triplicate. Aliquots of 100 mg of macerated seeds were extracted in plastic tubes with 2 ml of MeOH containing 0.1% (vol/vol) formic acid during 1 h in an ultrasonic bath. The extracts were filtered (0.22 μm), dried with a N_2_ flux, and stored at −20°C.

Seed extracts were resuspended in 1 ml of MeOH and aliquots of 100 μl and diluted with 900 μl and filtered through a 0.22-μm membrane. UHPLC-MS analyses were performed using a Thermo Scientific QExactive hybrid Quadrupole-Orbitrap mass spectrometer with the following parameters: electrospray ionization in positive mode, capillary voltage at 3.5 kV; capillary temperature at 300°C; S-lens of 50 V, and *m/z* range of 100.00 to 1,500.00. MS/MS was performed using normalized collision energy (NCE) of 20, 30, and 40 eV, and a maximum of 5 precursors per cycle were selected. A Waters Acquity UPLC BEH C18 1.7-μm (2.1 mm by 50 mm) column was used. Mobile phases were 0.1% (vol/vol) formic acid in water (A) and acetonitrile (B). Eluent profile (A:B) 0 to 10 min, gradient from 95:5 up to 2:98; held for 5 min; 15 to 16.2 min gradient up to 95:5; held for 3.8 min. The flow rate was 0.2 ml min^−1^. UHPLC-MS operation and spectrum analyses were performed using Xcalibur software (v.3.0.63). Samples were injected in random order. A quality control (QC) sample was prepared with 50 μl of each sample and was injected three times at the beginning of the batch and after three sample injections (47–49).

### Quantification of tryptoquialanine A.

Standard TA isolated from *P. digitatum* and EV extract were analyzed using a Waters Acquity UPLC system coupled to a Waters Micromass Quattro Micro TM API with electrospray ionization source and a triple quadrupole mass analyzer. Analyses were performed in the positive mode with an *m/z* range of 100 to 1,200, capillary voltage of 3 kV, cone voltage of 25 V, inlet capillary temperature of 150°C, and nebulizing gas temperature of 200°C. Stationary phase: Thermo Scientific column Accucore C18 2.6 μm (2.1 mm by 100 mm). Mobile phase: 0.1% formic acid (A) and acetonitrile (B). Eluent profile (A/B): 95/5 up to 2/98 within 10 min, held for 5 min, up to 95/5 within 1.2 min, and held for 3.8 min. The total run time was 20 min for each run, and the flow rate was 0.2 ml min^−1^. Injection volume: 10 μl. All the operation and spectrum analyses were conducted using Waters MassLynx v.4.1.

For the construction of the calibration curve, standard TA was diluted in the range concentration of 6.25 to 0.006 μg ml^−1^, and selected reaction monitoring (SRM) analyses were performed following conditions as previously described: *m/z* 519 → 197 (quantification) and *m/z* 519 → 213 (monitoring), collision energy of 22 eV (38).

For quantification of tryptoquialanine A in EVs, 40 μl of a 2.1 × 10^10^ EV ml^−1^ solution was dried and extracted with 100 μl of MeOH HPLC grade as previously described. Then, 100 μl of EV extract solution was transferred to glass vials and analyses were performed in duplicate.

### Statistical and metabolomic analyses.

Feature detection was performed on XCMS online (v.3.5.1) using the following parameters: method: centWave, prefilter peaks and intensity: 3 and 5,000, ppm: 2.5, Signal/noise threshold: 10, peak width: 5 to 20, mzdiff: 0.01, and noise filter: 1,000. Preprocessing included median fold change normalization on XCMS Online. Multivariate and univariate analyses of the feature list were performed with the MetaboAnalyst tool (v.4.0). Pareto scaling was applied. One-way analysis of variance (ANOVA) was performed, and all the results were analyzed using a confidence level of 95% and a significance level corresponding to *P < *0.05. Principal-component analysis (PCA) was performed for an exploratory analysis, followed by partial least-squares discriminant analysis (PLS-DA). A permutation test (cross validation) was performed to determine the reliability of the created PLS-DA model.

### Molecular networking analyses.

MS data were converted to mzXML format using MSConvert GUI, a tool of the ProteoWizard package. Molecular networks for *in vivo* assays and EV extracts were created using the mzXML files on the online workflow at the Global Natural Products Social Molecular Networking (GNPS) platform (http://gnps.ucsd.edu). Data were filtered by removing all MS/MS peaks within ±17 Da of the precursor ion. MS/MS spectra were window filtered by choosing only the top 6 peaks in the ±50-Da window throughout the spectrum. The data were then clustered with MS-Cluster with a parent mass tolerance of 0.02 Da and an MS/MS fragment ion tolerance of 0.02 Da to create consensus spectra. Consensus spectra that contained fewer than 2 spectra were discarded. A network was then created where edges were filtered to have a cosine score above 0.6 and more than 5 matched peaks. The spectra in the network were then searched against GNPS’s spectral libraries. The library spectra were filtered in the same manner as the input data. All matches between network spectra and library spectra were required to have a score above 0.6 and at least 5 matched peaks (50).
