# Supplementary figures and images for: Phytotoxic Tryptoquialanines Produced In Vivo by Penicillium digitatum Are Exported in Extracellular Vesicles
Source: mBio. 2021 Feb 9;12(1):e03393-20. doi: 10.1128/mBio.03393-20 (PMC7885104; doi:10.1128/mBio.03393-20)

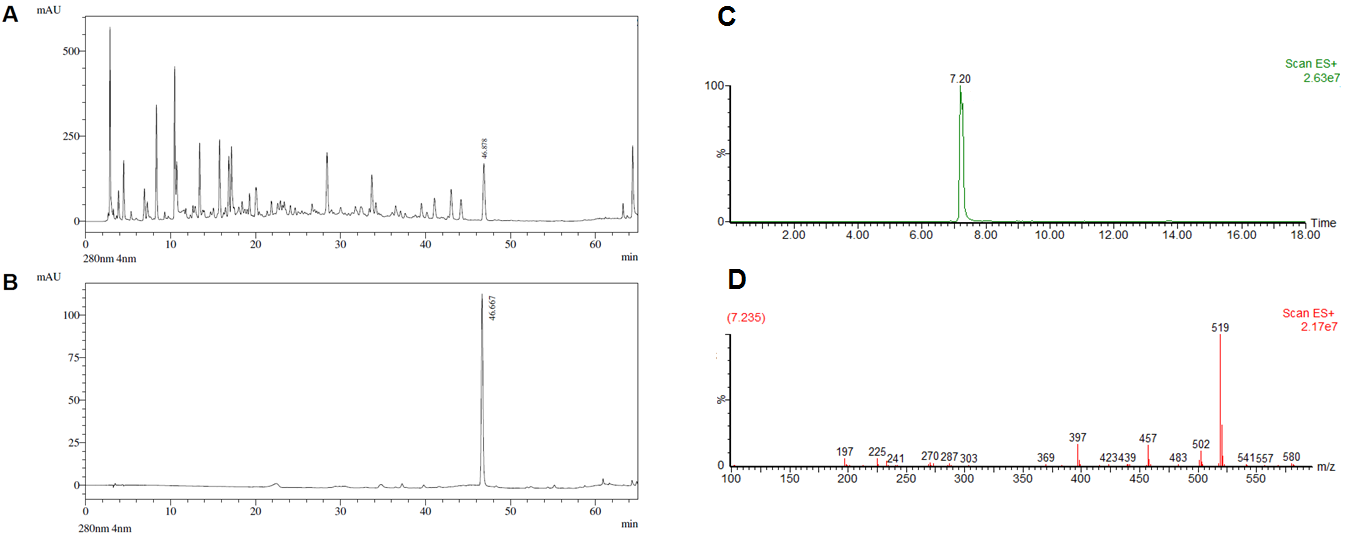

Supplement: FIG S1 [file mBio.03393-20-sf001.tif]

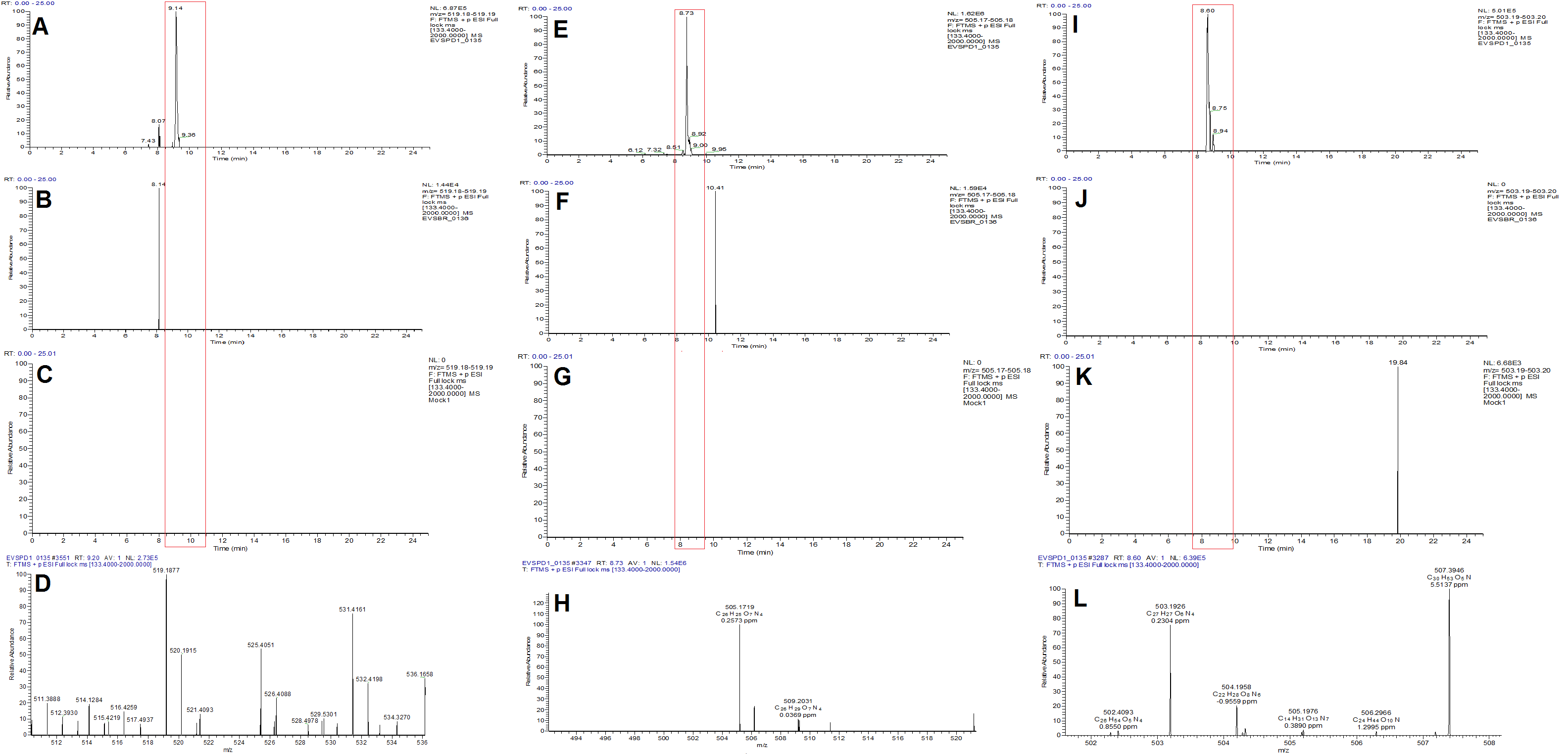

Supplement: FIG S2 [file mBio.03393-20-sf002.tif]

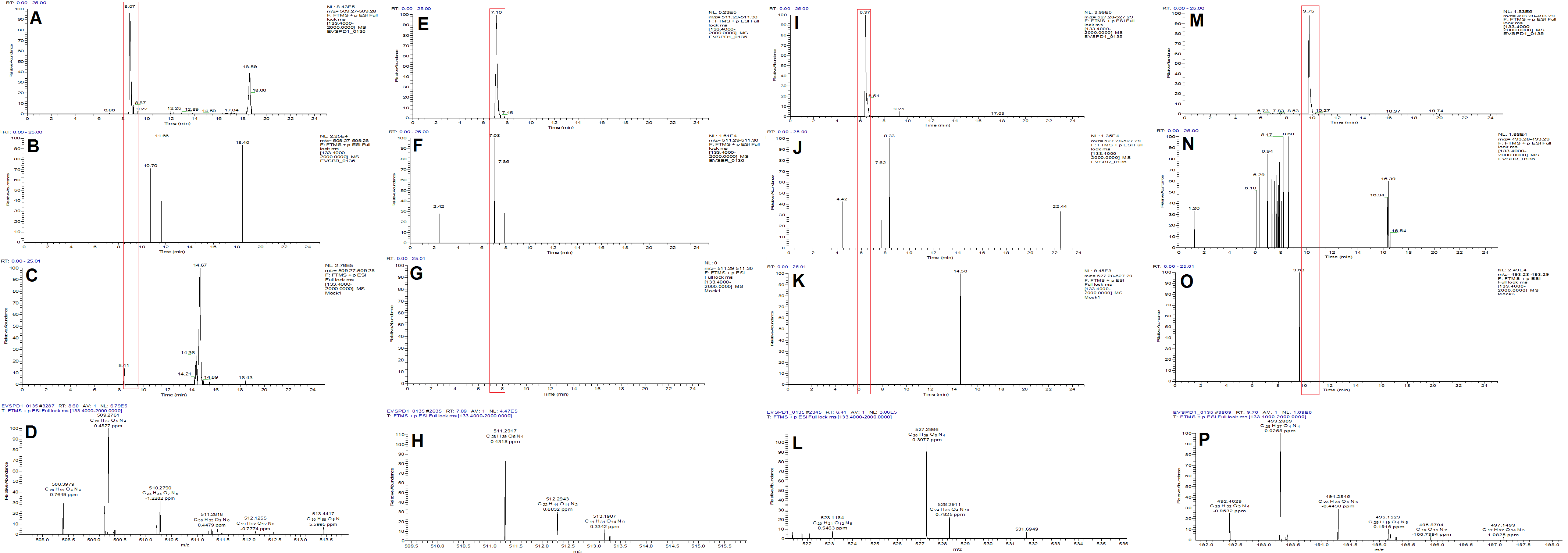

Supplement: FIG S3 [file mBio.03393-20-sf003.tif]

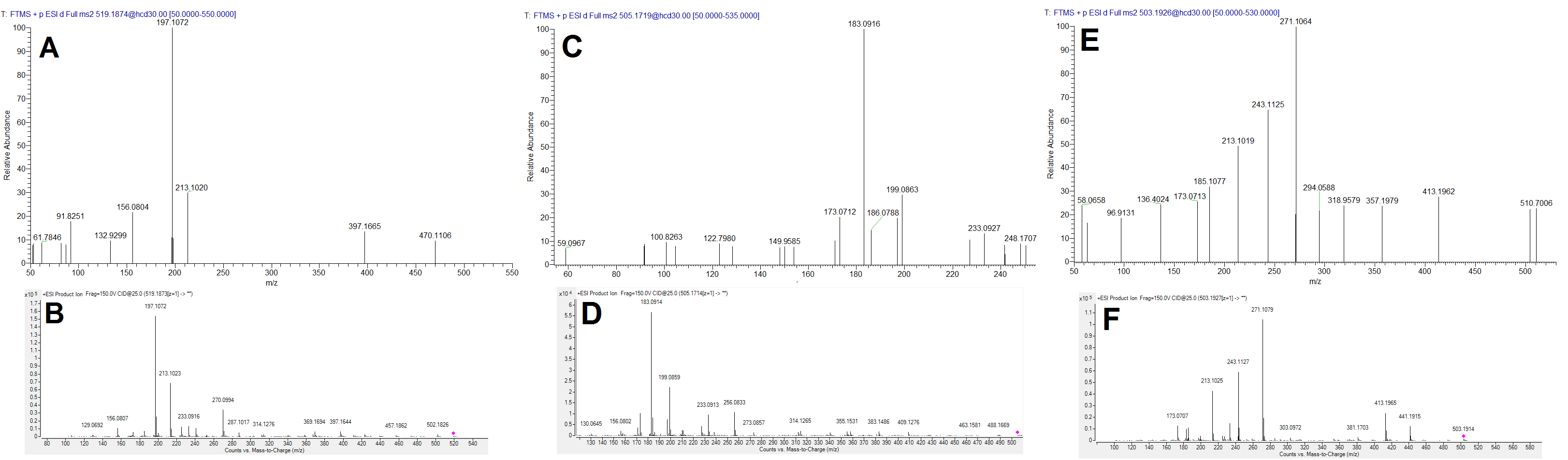

Supplement: FIG S4 [file mBio.03393-20-sf004.tif]

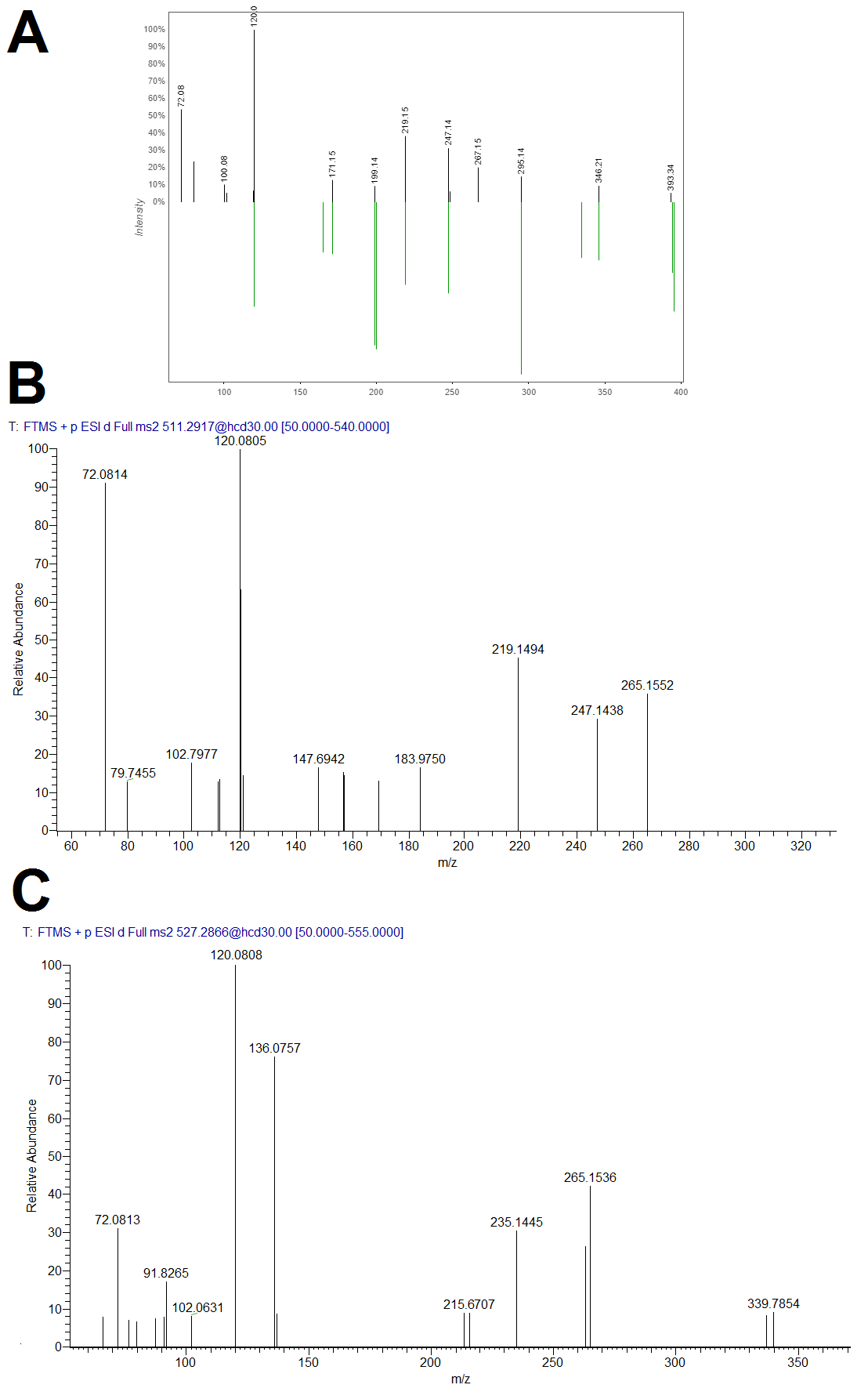

Supplement: FIG S5 [file mBio.03393-20-sf005.tif]

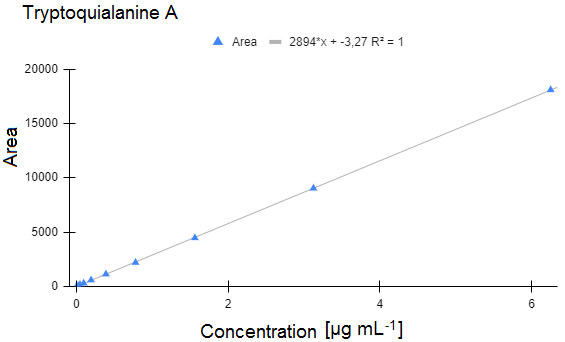

Supplement: FIG S6 [file mBio.03393-20-sf006.tif]

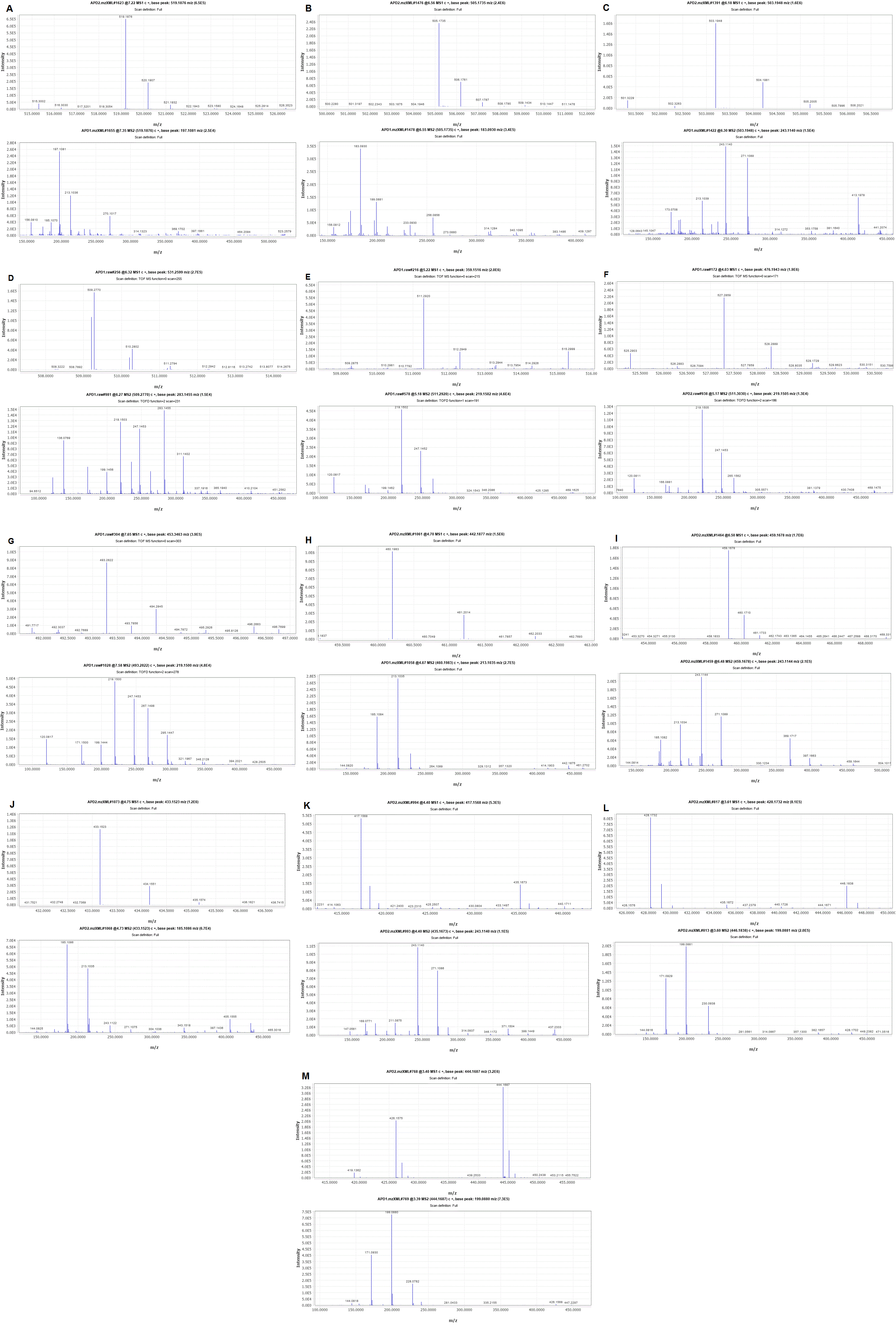

Supplement: FIG S7 [file mBio.03393-20-sf007.tif]

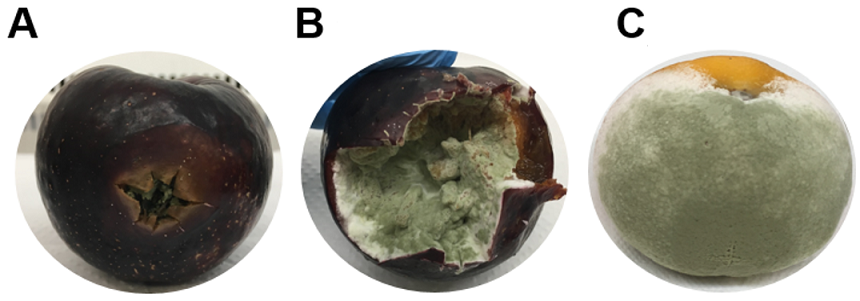

Supplement: FIG S8 [file mBio.03393-20-sf008.tif]

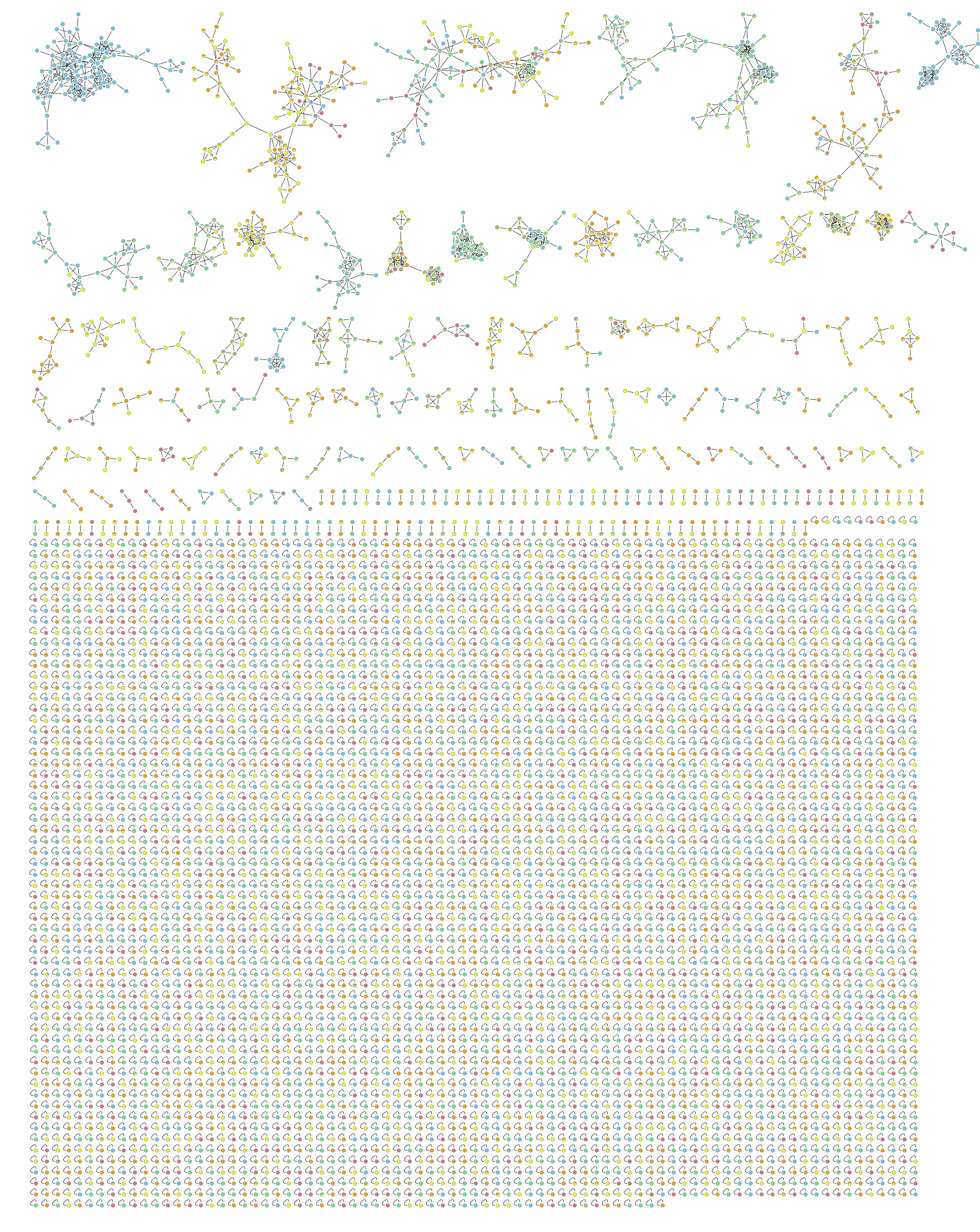

Supplement: FIG S9 [file mBio.03393-20-sf009.jpg]

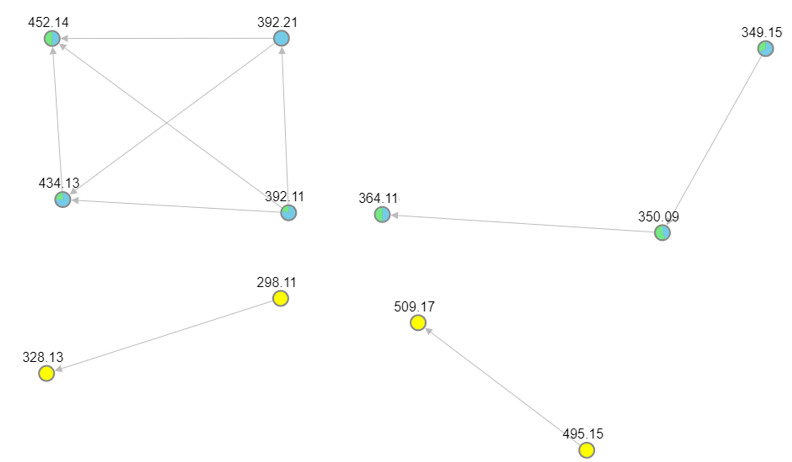

Supplement: FIG S10 [file mBio.03393-20-sf010.tif]
